# Genetic Variation in Functional Traits Influences Arthropod Community Composition in Aspen (*Populus tremula* L.)

**DOI:** 10.1371/journal.pone.0037679

**Published:** 2012-05-25

**Authors:** Kathryn M. Robinson, Pär K. Ingvarsson, Stefan Jansson, Benedicte R. Albrectsen

**Affiliations:** 1 Umeå Plant Science Centre, Department of Plant Physiology, Umeå University, Umeå, Sweden; 2 Department of Ecology and Environmental Science, Umeå University, Umeå, Sweden; University of California, United States of America

## Abstract

We conducted a study of natural variation in functional leaf traits and herbivory in 116 clones of European aspen, *Populus tremula* L., the Swedish Aspen (SwAsp) collection, originating from ten degrees of latitude across Sweden and grown in a common garden. In surveys of phytophagous arthropods over two years, we found the aspen canopy supports nearly 100 morphospecies. We identified significant broad-sense heritability of plant functional traits, basic plant defence chemistry, and arthropod community traits. The majority of arthropods were specialists, those coevolved with *P. tremula* to tolerate and even utilize leaf defence compounds. Arthropod abundance and richness were more closely related to plant growth rates than general chemical defences and relationships were identified between the arthropod community and stem growth, leaf and petiole morphology, anthocyanins, and condensed tannins. Heritable genetic variation in plant traits in young aspen was found to structure arthropod community; however no single trait drives the preferences of arthropod folivores among young aspen genotypes. The influence of natural variation in plant traits on the arthropod community indicates the importance of maintaining genetic variation in wild trees as keystone species for biodiversity. It further suggests that aspen can be a resource for the study of mechanisms of natural resistance to herbivores.

## Introduction

European aspen, *Populus tremula* L., is a pioneer tree valued as a host to many species of dependent flora and fauna, many of which are specialists [1]–[4]. Live and dead aspen trees alike harbour a variety of arthropods on the canopy and woody tissue [5]. Aspen is a common forage food for mammals [4] and also supports species of epiphytes [1], [2]. In recognition of this conservation value, current forestry practice in Scandinavia retains aspens in clear-cut zones to promote biodiversity [6]. In North America, the ecological, morphological and physiological characteristics of quaking aspen, *P. tremuloides*, have been extensively studied [7]–[13]. In contrast to *P. tremuloides*, there has been a relative paucity of knowledge of the same traits in *P. tremula*, however an interest in the European aspen has gradually emerged, recognising the genetic variation within this species [14], [15] in addition to its ecological value. In Sweden intra-specific variation for growth and phenological traits has been detected in young plants [16], [17]. Bylesjö et al [18] reported variation in leaf shape in the same population. Half-sib families of *P. tremula* have been shown to differ in growth and resistance to biotic stresses [19]. Recent chemical analyses of *P. tremula* leaves have isolated genotype-specificity in phenolic glycoside compounds [20] recognised for their efficacy in herbivore defence in *Populus* [21], however the response of folivorous arthropods has received little attention.

A growing body of evidence documents the influence of host-plant genotype on herbivore community structure. The concept of community genetics [22], [23], whereby genes conferring variation in plant traits in turn influence herbivore preferences as an extended phenotype, has been validated in a number of ecological systems, with clear examples in both inter-specific hybrids [24]–[27] and intra-specific hybrids [28]–[33]. The mechanisms of natural variation in herbivore preferences have been examined extensively and have been accounted for by traits including differences in stem growth [34], plant ontogeny [35]–[39], plant architecture [40], leaf biomass [41], environmental factors [42], secondary metabolites [43]–[45], physical defence [46], and factors in combination [47]–[48]. Secondary metabolites in leaf tissue, including phenolic compounds, are known to be under genetic control in trees [49], [50] and have been associated with the abundance of individual folivores and multi-trophic communities [49], [51]. Some specialist arthropod herbivores utilize plant phenolic compounds to elicit defences against predators [52], [53]. Although there are many such examples of coevolved defences in the on-going evolutionary battle between plants and their attackers, and many strategies of plant defence have been proposed and recycled [54]–[60], there is increasing awareness that a number of co-varying plant traits may be involved in the susceptibility to, and defence against, herbivores [61], [62].

Common garden trials have long been employed to provide the same set of environmental conditions to enable comparison of plant genotypes within an experimental population in herbs and trees [63], [64]. The Swedish Aspen Collection (SwAsp) was established in 2004 from the clonal propagation of 116 *P. tremula* trees collected from 12 regions across Sweden, spanning nearly ten degrees of latitude, and grown in two common gardens, one in northern and one in southern Sweden [17]. It is in this population that latitudinal genetic differentiation has been documented for several plant traits [16], [17], in addition to latitudinal trends of attack by rust fungus and leaf-mining insects that reflect countrywide forest survey data [65].

The ability of arthropods to resolve between plants within a species, interpreted through their survival, diet and oviposition preferences, has been observed in *Populus* species in controlled conditions [9], [66] and field studies [44], [67], [68] however we could not find any reported field observations of the total arthropod community in natural conditions in *P. tremula*. We examined the SwAsp collection to assess the range of intraspecific variation in plant functional traits that could influence dependent organisms. We conducted field studies initially in two common gardens to form an inventory of arthropod herbivores on the canopy of *P. tremula* in Sweden. We gathered arthropod data over two growing seasons in the common garden in northern Sweden and measured a suite of basic plant traits to address the following questions. (1) Are plant functional traits genetically and geographically structured in *P. tremula*? (2) Is the community of arthropod herbivores on *P. tremula* heritable? Identifying clonal differentiation in susceptibility to herbivores is informative both for conservation biology and to understand natural resistance to biotic stresses. Finally we ask (3) is the community of arthropod herbivores explained by specific plant traits?

## Materials and Methods

Common gardens were established in 2004 from root cuttings of mature trees collected from 12 regions across Sweden (Table 1), as detailed in Luquez et al. [17]. Briefly, one garden in Ekebo, southern Sweden (55.9°N) and one garden in Sävar, northern Sweden (63.9°N) were planted with at least four replicate trees of 116 clones. Sites were deer-fenced and weeded.

**Table 1:** Original locations of SwAsp Collection populations.

Location	Latitude	Longitude	Clone IDs
Arjeplog	66.19	18.43	101 - 91
Luleå	65.62	22.19	111 - 116
Dorotea	64.37	16.44	81 - 90
Umeå	63.93	20.63	91 - 100
Delsbo	61.73	16.70	71 - 80
Älvdalen	61.22	13.97	51 - 60
Uppsala	59.81	17.90	61 - 70
Brunnsberg	59.63	12.96	41 - 50
Vårgårda	57.99	12.93	21 - 30
Ydre	57.80	15.28	31 - 40
Simlång	56.71	13.25	1 - 10
Ronneby	56.27	15.21	11 - 20

Clones were produced from six parent trees from Luleå and ten parent trees at each other location, and assigned identity numbers (IDs), whereby increasing ID number indicates increasing latitude.

### Arthropod surveys

Arthropod surveys were undertaken in 2008 at the Ekebo common garden on 23–25 June and 15–16 July. Three surveys were made at Sävar in 2008 on 27–29 June, 7–9 July and 29–31 July, and in 2009 on 15 June, 7–9 July and 27–29 July. Arthropod loads differed between sites, with a paucity of arthropod abundance in Ekebo, where only two species accounted for over 75% of individuals counted. Therefore Ekebo data were excluded from our analyses, however the species inventory has been included in the supplementary information (Table S1) to ensure our records of canopy arthropods on aspen are as comprehensive as possible. Each tree was surveyed exhaustively by systematically examining all leaves on every branch and the types and numbers of arthropods recorded. Trees were surveyed only on sunny days and in varying order during different surveys to randomize any effects of timing or human disturbance on the fauna. Numbers or clusters of all leaf-dwelling arthropods, or leaf structures formed by arthropods, visible to the naked eye on every leaf were recorded, with the exception of two abundant galling mite species (*Aceria varia* and *Phyllocoptes populi*), which were scored once in the season by assigning an infection score (0, 0.5, 1, 2, 3, 4, or 5, where 5 = all leaves infected). Surveys were conducted by two individuals working together and counting methods were standardized before data was recorded. One author (KMR) undertook all surveys to ensure consistent scoring and counting of morphospecies. Arthropods were identified using field guides [69], [70]. Unknown arthropods were sampled and experts consulted on identification. It was necessary to classify some arthropods solely by the larval shelters (e.g. leaf rolls, ties and folds), leaf galls and subcutaneous mines constructed by the arthropods on the aspen leaves. All morphospecies are documented in reference material held at Umeå Plant Science Centre. Morphospecies were further categorized for some analyses by behaviour guild of each species based on how they utilize leaf tissue (leaf-gallers, -chewers, -miners and -rollers). Species richness was calculated as the sum of species on each individual plant. Arthropod abundance was the sum of all individuals of all arthropod species on each plant. Arthropod species known to feed on one plant genus (*Populus*) were defined as specialists, and all others considered generalists.

### Damage phenotyping

Infection by leaf rust fungus of the genus *Melampsora* was assessed visually on all individual plants at Sävar, assigning a score of 0 (absent), 1, 2, 3, 4, to 5 (every leaf infected) for the whole tree. This scoring took place in late August 2007 when among-tree differences in infection were highest and before autoinfection had spread to all trees [65]; at this time the scores in the field were poisson-distributed. All phenotyping on visual scales was repeated to ensure that scoring was consistent.

### Leaf and stem phenotyping

Stem heights and diameters were measured annually at the end of the growing season as described by Luquez et al. [17]. Leaf chlorophyll and anthocyanin contents were estimated indices using chlorophyll and anthocyanin content meters (Opti-sciences Inc, Hudson, USA). Readings were taken on five fully expanded, undamaged leaves chosen evenly across the plant on all individual plants. Leaves were sampled for morpho-chemical analyses immediately after the third survey of the season. Ten fully expanded, mature, undamaged leaves were selected from each plant and harvested at the petiole junction with the leaf. Samples were kept in cool conditions and returned to the laboratory. Whole leaves were scanned using a desktop scanner at 300 dpi. Petiole length was measured on the leaf scans using ImageJ [71]. Petioles were then erased using imaging software. Leaf images were measured using LAMINA software [18] to obtain leaf area. After scanning, leaves were air-dried at room temperature in well-ventilated conditions until constant weight, and then leaf dry weight was used to calculate specific leaf area (cm^2^ g^−1^). Dry leaf material was pooled per plant and ground using a Retsch bead mill for use in chemical analyses. Spring bud flush was scored every two days and the Julian date was recorded for an individual tree when a minimum of three leaves had burst their bud scales revealing a characteristic V-shape between the first two unfolding leaves.

### Leaf chemistry

Total phenolics and condensed tannins were quantified in the samples of dry, ground leaf tissue. Total phenolics were quantified from extracts in 80% methanol following the method of Ossipova et al. [72] against a chlorogenic acid standard (Sigma, St Louis, USA) and expressed as chlorogenic acid equivalents. Condensed tannins were quantified using the acid-butanol method using leaf powder extracted in 70% v/v acetone, 1% ascorbic acid [72] using a procyanidin-B standard (Sigma-Aldrich, St Louis, USA) and expressed as procyanidin equivalents.

### Statistical analysis

Statistical analyses were conducted in *R* [73]. Shannon diversity indices were calculated in PRIMER-E (version 6.1, Roborough, UK). Field blocks were tested for homogeneity of tree growth with two-way analyses of variance. There was no evidence of interaction between clone and block for stem height and diameter, from which we infer reasonable uniformity of growth conditions and randomization within the gardens. Arthropod morphospecies data did not conform to a normal distribution and could not be transformed to normality so Kruskal-Wallis analyses of variance were used to test between field blocks to confirm that the presence of morphospecies did not differ significantly between blocks. Data were transformed using Box-Cox powers where necessary. Box plot distributions were constructed, principal components analysis conducted, and latitudinal relationships were calculated from trait data from all individuals and clones. For all other analyses, clones with less than three live replicates were removed from the data set. Population differentiation (Q_ST_) and broad-sense heritability (H^2^) were calculated as in Luquez et al. [17]. Briefly, we used data from the common garden design to estimate the variance among clones within a population and among populations. For estimating the heritability of the traits we used the following linear model:
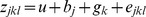
where z_jkl_ is the phenotype of the *lth* individual in the *kth* block from the *jth* clone and where u denotes the grand mean and e_jkl_ is the residual error term. Broad-sense heritability was calculated from estimates of total genetic variation (σ_G_^2^) and total phenotypic variation (σ_P_^2^)
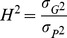
To estimate Q_ST_ we used:
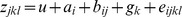
Here the population (a_i_) and clone (b_ij_) effects now provide estimates of genetic variation between populations (σ_B_^2^) and among clones within populations (σ_C_^2^), respectively. These variance components were used to estimate Q_ST_ according to:
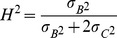
A Bayesian approach was used to calculate point estimates and confidence regions of Q_ST_, implemented in the R-package *MCMCglmm* [74]. For each trait a single MCMC chain was run for 15000 steps, with the first 5000 discarded as burn in. The remaining samples were thinned by taking every tenth sample, yielding 1000 independent draws from the posterior distribution of the parameters of the model. Convergence of the chains was assessed using the *coda* package in *R*. The priors of the variances and covariances of the random effects in the model are locally non-informative priors [74]. Point estimates of H^2^ and Q_ST_ were calculated as the mode of the posterior distributions and confidence intervals were calculated from 95% highest posterior density (HPD) regions. As with all broad-sense heritability calculations, our estimates of H^2^ and Q_ST_ are likely to be over-estimates of true heritabilities [75], however these data enable the comparison of the genetic and environmental components of the traits measured.

Arthropod data were classified into motile species that could move between plants, and sessile species that select the host plant for feeding or oviposition indicated by a modification in the leaf tissue. For community analysis we defined ‘arthropod community’ as these sessile leaf-modifying species to avoid ambiguity of their relationship with plant. For other analyses we used data from all morphospecies. For community analysis we used the *vegan* package in *R* [76]. Bray-Curtis similarity matrices were constructed from all leaf-modifying arthropod traits in each year. Prior to this, count data were square root transformed to reduce differences between common and rare species and all data were then normalized. Data from each year were analyzed separately using the *adonis* function in the R package *vegan*, a non-parametric multivariate analysis of variance (non-parametric MANOVA), which partitions the dissimilarity matrix (the response variable) by fitting linear models (with genetic, population and plant traits as explanatory variables), and permuting the data within the replicated field blocks to return pseudo F-values on 999 permutations [77]. This method makes few assumptions about the underlying data structure and is thus considered suitable for the analysis of community count data and when the response variable is comprised of metric (Euclidian) distances the F-value is identical to that obtained using parametric ANOVA [77]. Principal components analysis (PCA), an ordination method maximizing the differences between individuals, was conducted using *R* software on all clones (irrespective of replication). Stem diameter was excluded from the PCA analyses because it is highly correlated to stem height (Pearson correlation: *r* = 0.9, *P*<0.001).

## Results

**Figure 1:**
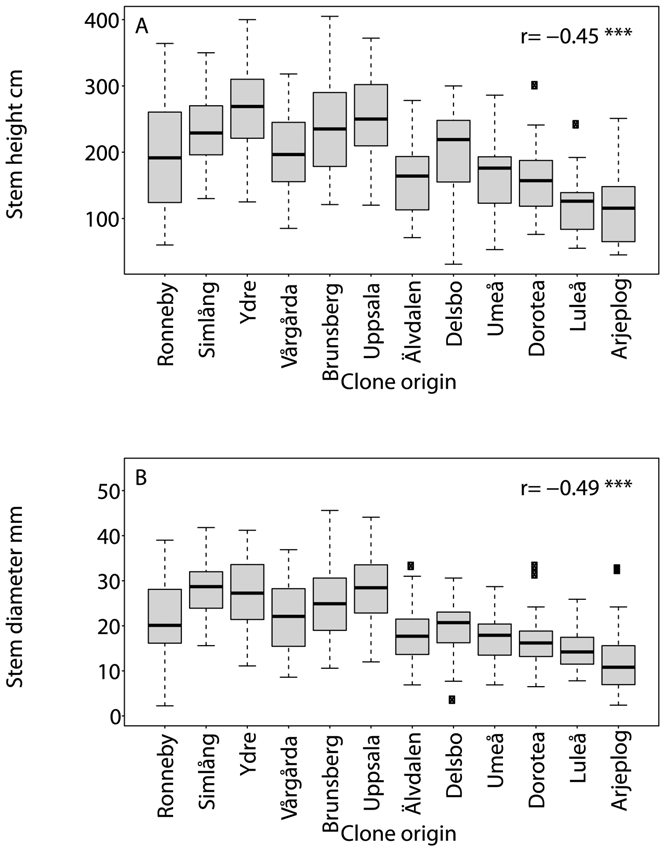
Stem heights and diameters of SwAsp clones grouped by population. Stem heights (A) and diameters (B) of all SwAsp clones grouped by their population of origin, ranked from south to north, in the Sävar common garden in 2009. Six to 10 clones are represented per location. Spearman rank coefficient, *r*, indicates correlation between trait and latitude of population origin; *** indicates statistical significance at *P*<0.001.

**Figure 2:**
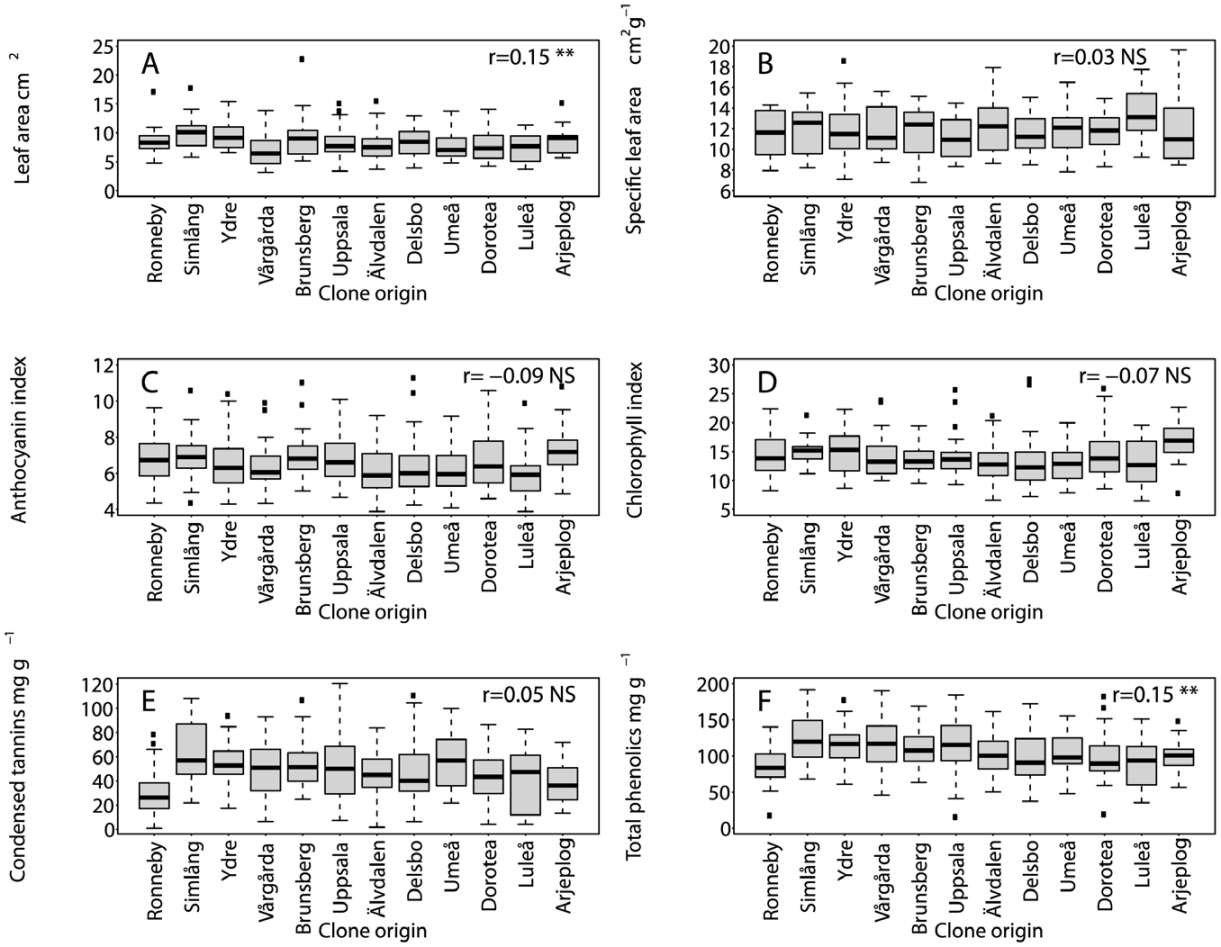
Leaf trait values of SwAsp clones grouped by population. Distribution of leaf trait values of all SwAsp clones grouped by their population of origin, ranked from south to north. Traits were measured in the Sävar common garden in 2008 (A, B, C, D) and 2009 (E, F). Six to 10 replicated clones are represented per location. Spearman rank correlation coefficients, *r*, between trait and latitude of clone origin are shown with the following significance levels: ** *P*<0.01, and *NS* non-significant.

Stem traits followed a latitudinal gradient when evaluated at the population level (Figure 1), with the tallest trees of largest girth originating from the southern part of Sweden. These regional differences in stem biomass are indicated in Q_ST_ values (Table 2), with clear population differentiation for stem height and diameter, but also a high level of within-clone similarity indicated by the H^2^ values (Table 2). In contrast, leaf morphs (Figure 2) did not show strong latitudinal trends. Despite negligible Q_ST_ values for leaf traits, H^2^ values were moderate to high for all plant traits measured except specific leaf area (which had no apparent heritable component), suggesting abundant genetic variation but little genetic differentiation for these traits.

**Table 2:** Population differentiation (Q_ST_) and broad-sense heritability (H^2^) of stem, leaf and arthropod community traits in the SwAsp common garden, Sävar (63°54′N).

Trait	Year measured	Confidence interval (Q_ST_)	*Q*_ST_	Confidence interval (H^2^)	H^2^
Stem height	2008	0.22–0.75	**0.44**	0.38–0.58	**0.47**
Stem height	2009	0.25–0.76	**0.50**	0.46–0.64	**0.56**
Stem diameter	2008	0.47–1.00	**0.61**	0.31–0.52	**0.39**
Stem diameter	2009	0.31–0.81	**0.54**	0.38–0.58	**0.49**
Leaf area	2008	0.00–0.33	**0.00**	0.11–0.39	**0.25**
Specific leaf area	2008	0.00–0.92	**0.02**	0.00–0.08	**0.00**
Petiole length	2008	0.00–0.24	**0.01**	0.32–0.63	**0.48**
Chlorophyll index	2008	0.00–0.16	**0.00**	0.27–0.48	**0.41**
Anthocyanin index	2008	0.00–0.18	**0.00**	0.20–0.42	**0.29**
Condensed tannins	2009	0.00–0.26	**0.00**	0.45–0.57	**0.55**
Total phenolics	2008	0.00–0.29	**0.00**	0.12–0.36	**0.24**
Total phenolics	2009	0.00–0.28	**0.00**	0.27–0.49	**0.36**
Date of budflush	2011	0.00–0.17	**0.02**	0.61–0.77	**0.69**

Calculations are based on clones with data for three or more live replicates per clone.

**Figure 3:**
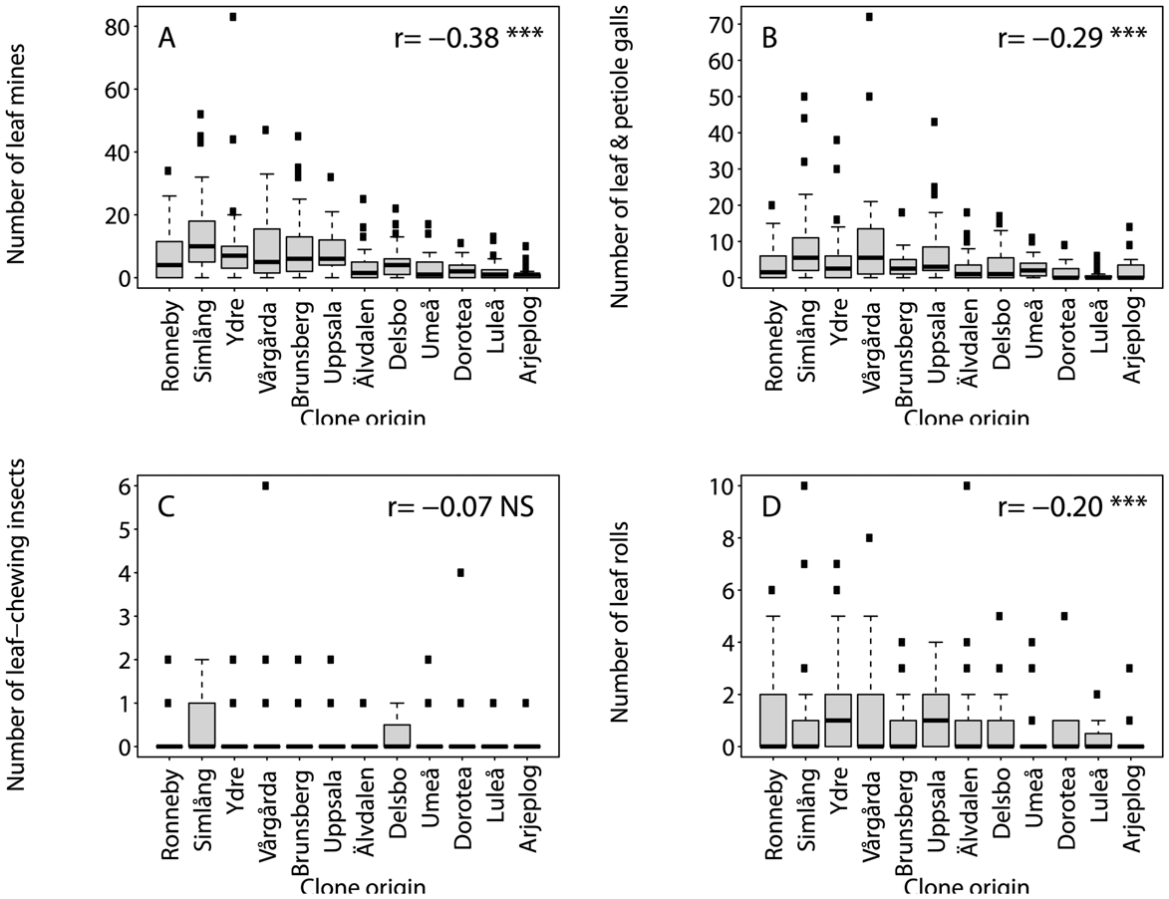
Numbers of arthropods categorised by feeding guilds recorded on SwAsp clones grouped by population. Numbers of arthropods grouped by feeding guilds recorded on all SwAsp clones grouped by their population of origin, ranked from south to north. Traits were measured at maximum abundance in 2009 in the Sävar common garden. Six to 10 replicated clones are represented per location. Spearman rank correlation coefficients, *r*, between trait and latitude of clone origin are shown with the following significance levels: *** P<0.001, ** P<0.01.

In total 93 arthropod morphospecies (Table S1) were recorded on *P. tremula* in the SwAsp common gardens. The most frequent arthropods were specialists on *Populus*, including many that cause galls or make rolls, ties, mines and other shelters using aspen leaves (Table 3). Most arthropod species increased in frequency in the common garden from 2008 to 2009. Arthropods grouped by guilds were significantly and negatively correlated with latitude (Figure 3); the strongest relationship (r = −0.38) was for leaf-mining insects. Leaf-mining, gall-making and leaf-rolling arthropods each showed stronger correlations with latitude than any leaf trait, but weaker correlations than stem traits. Leaf-chewing arthropods were rare (Figure 3). Table 4 shows heritability and population differentiation for leaf rust fungus and arthropod traits in the common garden. It was not possible to calculate Q_ST_ or H^2^ on individual morphospecies due to the small number of individuals in the data set, however when all arthropod (arthropod abundance) or species (species richness) were summed on an individual plant there were sufficient data to calculate Q_ST_ and H^2^ on the 2009 data set. Arthropod species richness and abundance were moderately heritable, but less so than infection by leaf rust fungus, which showed a strong population and clonal heritability (Table 4). Q_ST_ was generally comparable to values obtained for stem traits in contrast to the negligible or absent Q_ST_ values for leaf traits. Furthermore, the Shannon diversity index, despite showing little genetic variation, showed Q_ST_ values indicative of a significant degree of aspen population structure. Non-parametric MANOVA (Table 5) identified genotypic and population differences in the distance matrix of the arthropod community in both years.

**Table 3:** The most common arthropod morphs in the SwAsp common garden at Sävar (63°54′N), expressed as the maximum percentage of the trees in the garden affected by each species.

				Maximum % trees affected	
Leaf-feeding guild	Arthropod species/morph	Action on leaf	Host specificity	2008	2009
Gall-maker	*Aceria varia*	Mite causes red-brown galls appearing as spots with a granular texture.	Specialist	30.98	42.93
Gall-maker	*Contarinia petioli*	Midge larva causes petiole gall, 5–10 mm diameter.	Specialist	9.76	23.50
Gall-maker	*Dasineura populeti*	Midge larva infests leaf edges causing distinctive upward curl of blotchy tissue.	Specialist	3.41	15.98
Gall-maker	*Eriophyes diversipunctatus*	Mite infestation of leaf blade/petiole junction causing red swellings.	Specialist	1.22	0.48
Gall-maker	*Harmandia cavernosa*	Midge larva causes gall to form on both sides of the leaf plane.	Specialist	1.46	13.67
Gall-maker	*Harmandia gobuli*	Yellow-red larva causes elongated gall, 2–3 mm diameter, on adaxial leaf surface.	Specialist	2.44	11.86
Gall-maker	*Harmandia tremulae*	Midge larva causes round galls on adaxial leaf surface, 3–6 mm diameter.	Specialist	0.73	1.45
Gall-maker	*Phyllocoptes populi*	Mite causes pale green galls which appear as dents on adaxial leaf surface.	Specialist	20.49	42.34
Leaf-chewer	*Caliroa tremulae* ^§^	Sawfly larva chews leaf tissue.	Specialist	0.24	3.39
Leaf-chewer	*Phratora vitellinae* ^§^	Adult beetle oviposits on and chews tissue.	Generalist	8.29	38.50
Leaf-chewer	*Phyllobius maculicornis* ^§^	Adult weevil chews leaf tissue.	Generalist	20.00	11.51
Leaf-miner	*Aulagromzya tremulae*	Larva mines beneath abaxial epidermis leaving a yellow trail.	Specialist	19.02	12.35
Leaf-miner	*Phyllocnistis labyrinthella*	Larva mines beneath abaxial epidermis leaving grey frass line.	Specialist	36.10	75.18
Leaf-miner	*Phyllocnistis unipunctella*	Larva mines beneath adaxial epidermis leaving iridescent white frass line.	Specialist	19.02	33.58
Leaf-roller	*Archips* spp. (lepidopteran rolls)	Larva rolls single or multiple leaves parallel with, or perpendicular to, the midrib.	Generalist	14.63	44.07
Leaf-roller	*Byctiscus betulae/Byctiscus populi*	Imago punctures petiole and rolls the resulting wilted leaf roll to oviposit in.	Generalist/Specialist	8.29	13.43
Leaf-tier	*Tethea or*	Larva ties two leaves together with silk to form a ‘sandwich’-like shelter.	Specialist	8.78	7.26

Specialist species are those known to feed/oviposit on only *Populus*, generalists on more than one genus. The one leaf-tying species was classified with the most-related guild, leaf-rollers, for the analysis. All arthropods construct obvious, fixed leaf structures unless indicated by ^§^, in which case the species is free living throughout its life cycle.

**Table 4:** Population differentiation (Q_ST_) and broad-sense heritabilities (H^2^) of biotic traits: *Melampsora* leaf rust infection, and arthropod herbivore community traits in the SwAsp common garden at Sävar (63°54′N).

Trait	Year	Confidence interval (Q_ST_)	*Q*_ST_	Confidence interval (H^2^)	H^2^
Leaf rust	2007	0.24–0.74	**0.54**	0.39–0.59	**0.47**
Arthropod abundance	2009	0.08–0.56	**0.23**	0.31–0.52	**0.38**
Species richness	2009	0.19–0.77	**0.40**	0.25–0.47	**0.36**
Shannon index	2009	0.18–0.91	**0.42**	0.08–0.28	**0.15**

Calculations are based on clones with data for three or more live replicates per clone.

**Table 5:** Differentiation of arthropod community composition by plant genotype and population in the Sävar common garden (63°54′N).

Garden	Source	d.f.	*SS*	*MS*	*F*	*P*
Sävar 2008	Genotype	95	13.655	0.14374	1.2587	**0.03**
	Residual	273	31.176	0.1142		
	Total	368	44.831			
	Population	11	2.57	0.23367	1.974	**0.01**
	Residual	357	42.26	0.11838		
	Total	368	44.831			
Sävar 2009	Genotype	97	43.384	0.44726	118.73	**0.001**
	Residual	278	1.047	0.00377		
	Total	375	44.431			
	Population	11	39.506	3.5914	265.41	**0.001**
	Residuals	364	4.925	0.0135		
	Total	375	44.431			

Non-parametric MANOVA tests were conducted on a Bray-Curtis dissimilarity matrix of sessile, leaf-modifying arthropod species with 999 permutations stratified by field block.

**Figure 4:**
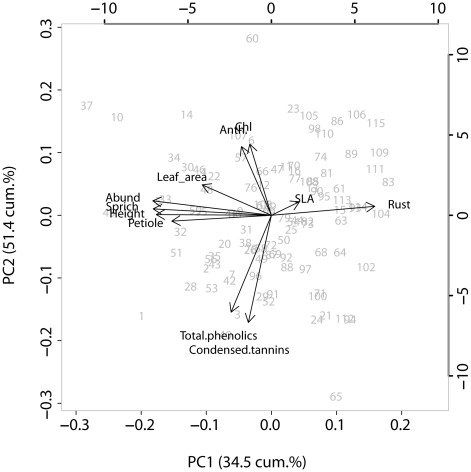
Principal components biplot of plant and arthropod traits in Sävar common garden. Biplot of the first two components of principal components analysis conducted on stem and leaf trait with arthropod abundance and species richness in the SwAsp common garden at Sävar. The first and second principal components respectively represent 34.5% and 51.4% of the total variation explained. Analysis was conducted on all clones, labelled as grey numbers from the populations indicated in Table 1. Trait abbreviations: Petiole = petiole length, Anth = anthocyanin index, Chl = chlorophyll index, SLA = specific leaf area, Abund = arthropod abundance, Sprich = species richness.

The first two components from the principal components analysis (PCA) explained 51.4% of the total variation in the aspen phenotypes (Figure 4). The first principal component (PC1) was influenced by plant height, arthropod species richness and abundance, together with petiole length and to a lesser extent leaf area (Table 6). Leaf rust fungus influenced PC1 such that smaller trees were more heavily infected (as shown in [65]). Leaf traits accounted for most variation in PC2, with contrasting trait loadings of secondary chemical compounds and anthocyanin and chlorophyll indices (Table 6).

**Table 6:** Trait loadings of the first (PC1) and second (PC2) principal components of analysis on plant traits and arthropod indices in 2009, with PC1 explaining 34.5% and PC2 explaining 51.4% of the cumulative variation in the data set.

Trait	PC1 loading	PC2 loading
Arthropod abundance	−0.44	0.08
Species richness	−0.44	0.04
Height	−0.43	0.00
Petiole length	−0.37	−0.03
Leaf area	−0.26	0.17
Total phenolics	−0.15	−0.54
Anthocyanin index	−0.11	0.38
Condensed tannins	−0.09	−0.60
Chlorophyll index	−0.08	0.40
Specific leaf area	0.11	0.08
Leaf rust infection	0.39	0.05

Non-parametric MANOVA tests were used to test plant traits as factors explaining arthropod community distance matrices. Table 7 shows results for 2009, indicating that stem height, stem diameter, leaf area, petiole length and anthocyanins, each individually explained variation in arthropod community composition. However, specific leaf area, condensed tannins, total phenolics, and chlorophyll content did not appear to influence community composition in 2009. These results were reflected in non-parametric MANOVA tests with plant traits in the 2008 data set, where height (F = 1.83, *P* = 0.048), leaf area (F = 10.18, *P* = 0.001), petiole length (F = 5.94, *P* = 0.012), anthocyanins (F = 23.55, *P* = 0.001) and condensed tannins (F = 58.94, *P* = 0.001) each showed significant explanation of the variance for the arthropod community matrix, but specific leaf area, total phenolics and chlorophyll content (a proxy for leaf N) did not. The H^2^ of spring budflush date (0.69) suggested that this trait had enough of a genetic component to be tested against data from previous seasons, and despite nearing a significant result in the np MANOVA (Table 7), we cannot conclude that it explained variance in either arthropod community data set and the relationship between arthropod community and tree height is not a result of differences in growing season length.

**Table 7:** Differentiation of arthropod community composition in 2009 by plant functional traits.

Trait	Source	d.f.	*SS*	*MS*	*F*	*P*
Height	Stem height	1	3.133	3.13274	28.37	**0.001**
	Residual	374	41.299	0.11042		
	Total	375	44.431			
Diameter	Stem diameter	1	4.077	4.077	37.785	**0.001**
	Residual	374	40.354	0.1079		
	Total	375	44.431			
Individual leaf area	Individual leaf area	1	1.267	1.26692	10.579	**0.001**
	Residual	298	35.687	0.11975		
	Total	299	36.954			
Specific leaf area	Specific leaf area	1	0.714	0.7142	5.7512	0.596
	Residual	288	35.765	0.12418		
	Total	289	36.479			
Petiole length	Petiole length	1	0.7978	0.79777	6.4401	**0.002**
	Residual	233	28.8633	0.12388		
	Total	234	29.6611			
Condensed tannins	Condensed tannins	1	0.217	0.21711	1.7714	0.484
	Residual	351	43.018	0.12256		
	Total	352	43.235			
Total phenolics	Total phenolics	1	0.361	0.3607	2.9529	0.641
	Residual	351	42.874	0.12215		
	Total	352	43.235			
Chlorophyll index	Chlorophyll index	1	0.169	0.16863	1.3882	0.681
	Residual	354	43.002	0.12147		
	Total	355	43.171			
Anthocyanin index	Anthocyanin index	1	2.843	2.84314	24.957	**0.001**
	Residual	354	40.327	0.11392		
	Total	355	43.171			
Julian days to budflush	Julian days to budflush	1	0.115	0.11507	0.92848	0.079
	Residual	339	42.013	0.12393		
	Total	340	42.128			

Non-parametric MANOVA tests were conducted on a Bray-Curtis dissimilarity matrix of sessile, leaf-modifying arthropod species with 999 permutations stratified by field block.

## Discussion

### Genetic and geographic variation in plant traits

We found that plant functional traits including stem size, leaf morphology and basic leaf secondary chemistry, are heritable in *P. tremula*. Population and clonal variation in stem height and diameter indicates that the SwAsp collection has continued to exhibit the latitudinal clines in stem traits reported in the years directly after its establishment [16], [17]. Broad-sense heritability for stem and most leaf traits indicated strong differences between clones. This confirms recent observations of genetic variation in full-sib families of *P. tremula* for leaf phenolics and stem biomass [19] and stem traits in quaking aspen [11], [12]. In accordance with other studies in *P. tremula* we have confirmed a genetic component to individual leaf area [18], [78], which has also been observed in *P. tremuloides* [12], [79]. It has been found in *Populus trichocarpa*×*P. deltoides* hybrids that petiole length is a trait under the control of a few major genetic loci [80]. While there are weak correlations between tree height and petiole length in the SwAsp collection and in hybrid poplars [81], Wu et al. [82] found no genetic correlation between these two traits. Our data also show high levels of broad-sense heritability (H^2^ = 0.69) for spring bud flush phenology, reporting higher values than previously recorded in SwAsp [17] and other studies in hybrid poplars [83], [84]. The variation in these plant traits defines the range of living environments and food quality selected by arthropod herbivores.

### Genetic and geographic variation in canopy arthropods

Surveys indicated that *P. tremula* in Sweden supports a wide array of arthropod herbivores, and many of these species are specialists on aspen. This knowledge considerably enhances existing documentation of the canopy arthropod fauna on *P. tremula* and confirms the status of aspen as an important keystone species for biodiversity [3]–[5]. We discovered genetic variation in the arthropod assemblage in both 2008 to 2009, alongside an increase in the number of species and the proportion of trees affected by these species. We also observed differences in community composition between the populations of aspen. Earlier reports have identified heritability in community composition in *Populus* species but in smaller collections of genotypes than ours [33], [49]. Here we report heritability values for arthropod abundance, species richness and diversity (Shannon index), traits that differ between regional populations, suggesting that there is regional variation in resistance to canopy arthropods in accordance with our previous work on leaf-mining moths and leaf rust fungus [65].

### Plant traits influencing the arthropod community

We analyzed sessile leaf-feeding and leaf-dwelling arthropods and expected the assemblage of arthropod herbivores, whose relationship with the leaf is intimate and dependent on leaf tissue throughout one or more life-cycle stages, to vary in accordance with the leaf characteristics of plants that they select. Using principal components analysis we have shown that petiole length and leaf area are the next most important traits influencing the PC1 (Table 6) after tree height, arthropod abundance and species richness. The strong relationships between petiole length and individual leaf area with community composition indicates that variation in these traits likely influences the choice of host leaf tissue, especially given that these leaf-dwelling arthropods inhabit individual leaves or petioles during a vulnerable developmental stage [85], [86]. For this reason it is surprising that we have not identified a relationship between herbivores and specific leaf area [87]. It is also likely that larger leaves and petioles are chosen by herbivores for providing a greater amount of food. The weak relationship (Pearson correlation *r* = 0.123, *P* = 0.03) between chlorophyll content and leaf area indicates that leaf nitrogen content is not the basis of the relationship between leaf area and arthropod community.

We measured traits representing chemical resistance (total phenolics, condensed tannins and anthocyanins) and plant quality (leaf area, specific leaf area and chlorophyll, a proxy of leaf nitrogen content [88]). In contrast to previous reports in European [65] and trembling [44], [66], [89] aspen where herbivore damage was related to concentrations of phenolic glycosides, we identified no such correlation with total phenolics in our data from either year. We suggest that because most species in our surveys are specialists on *Populus* and well adapted to a diet rich in phenolic glycosides, they are not deterred by, and may benefit from [52], [53] leaf secondary chemistry as would be expected for generalists. This study investigated pooled classes of phenolic compounds rather than, for example, individual phenolic glycosides known to influence particular herbivores on *Populus* [66], [21]. Further analysis of specific phenolic defence compounds, rather than total phenolics and total condensed tannins, could confirm or refute this suggestion, and recent work reports a wider range of phenolic defence compounds in *P. tremula* than was previously detected [20]. We did identify significant partitioning of the variation in community composition by condensed tannins (in 2008) and anthocyanins (in both 2008 and 2009), both of which are non-structural compounds with common precursors in the phenylpropanoid pathway [90]. While much work is underway to dissect the relationships between the different compounds and their synthesis in this pathway [91], [92], our understanding of the biochemical and functional connection between condensed tannins and anthocyanins is not complete. However, it is from this phenylpropanoid pathway that the secondary metabolites affecting palatability of leaf tissue to arthropods are produced [92].

Numerous factors dictate the nutritive quality of plant tissue for herbivores. The products of primary metabolism can be allocated to the accumulation of biomass or the production of defensive compounds, or to both processes [59]. This balance has long been the subject of theoretical discussion [56], [57], [59], [60]. The variance in our herbivore community data explained by tree size, which is a function of growth rate in common garden plants of identical age, and the similarity of herbivore data to population structure for tree size, suggest that allocation of resources to growth is highly relevant to the herbivore community. This is consistent with other reports of the relationship between herbivore performance and plant growth [93]–[95]. However our results also indicate that the availability of leaf surface (leaf area and petiole length) and leaf nutritive quality (anthocyanins and condensed tannins) at this stage in development, is a significant influence in this arthropod community dominated by specialists.

Differences between developmental and ontogenetic stages in *Populus* physiology and morphology have been reported [12], [13], [96]. In accordance with a recent meta-analysis [97] we expect that in future surveys of the SwAsp trees, changes in response to herbivory would be punctuated by defined developmental stages, in particular terminal differentiation and sexual maturity, and these changes may be projected onto the arthropod community. We agree with Boege and Marquis [98] that a complete picture of the mechanisms of response to herbivores cannot be extrapolated from one ontogenic stage.

Having identified substantial intraspecific variation in several traits, we require an understanding of the mechanisms influencing these relationships in order to ascertain the impact of European aspen as a component of wider ecological systems. Genetic diversity within plant populations and plant communities has been shown to have ecological impact on consumer communities and wider ecosystem effects [89], [90], although we have yet to detect whether genetic variation in *P. tremula* can influence multi-trophic communities beyond the arthropod herbivore community as found in other *Populus* species [49], [51]. Our identification of nearly 100 arthropod morphs in the canopy of young *P. tremula* plants strengthens the evidence that genetic variation in aspen is important for maintaining biodiversity, particularly of specialist arthropods. We conclude that geographic and genetic factors, in particular plant vigour and leaf quality, structure the projection of phenotypic variation in young aspen trees to the herbivore communities that they host. Furthermore, these patterns reveal natural variation in susceptibility to insects, a useful resource as we seek mechanisms that could be deployed in breeding trees for herbivore resistance.

## Supporting Information

Table S1 Arthropod morphospecies recorded in the SwAsp common gardens at Sävar and Ekebo.
(DOCX)
