# Supplementary material for: Genetic Variation in Functional Traits Influences Arthropod Community Composition in Aspen (Populus tremula L.)
Source: PLoS One. 2012 May 25;7(5):e37679. doi: 10.1371/journal.pone.0037679 (PMC3360762; doi:10.1371/journal.pone.0037679)
Supplement: Table S1 — Arthropod morphospecies recorded in the SwAsp common gardens at Sävar and Ekebo. (DOCX) [file pone.0037679.s001.docx]

**Supporting Table S1.** Arthropod morphospecies recorded in SwAsp common gardens at Sävar and Ekebo.

| Arthropod | Order | Family | Species | Guild |
| --- | --- | --- | --- | --- |
| Ant | Hymenoptera | Formicidae | sp.1 | Visitor |
| Aphid (woolly) | Hemiptera | Aphididae | sp.1 | Sap-sucker |
| Aphid (waxy, dome-galler) | Hemiptera | Aphididae | sp.1 | Sap-sucker |
| Beetle (brown) | Coleoptera | Crepidodera | sp.1 | Visitor |
| Beetle (slim, brown) | Coleoptera | Oedemeridae | sp.1 | Visitor |
| Bilateral leaf galler | Diptera | Cecidomyiidae | *Harmandia cavernosa* | Gall-maker |
| Caterpillar (black/white) | Lepidoptera |  | Sp.1 | Leaf-chewer |
| Caterpillar (black/white/orange) | Lepidotera | Lymantriidae | *Leucoma salicis* | Leaf-chewer |
| Caterpillar (brown, furry) | Lepidoptera |  | sp.2 | Leaf-chewer |
| Caterpillar (brown, smooth) | Lepidoptera | Geometridae | sp.1 | Leaf-chewer |
| Caterpillar (furry black/white/red) | Lepidoptera | Noctuidae | *Acronicta rumicis* | Leaf-chewer |
| Caterpillar (geometrid, green) | Lepidoptera | Geometridae | sp.2 | Leaf-chewer |
| Caterpillar (green/beige) | Lepidoptera |  | sp.4 | Leaf-chewer |
| Caterpillar (green, hairy, tiny) | Lepidoptera |  | sp.5 | Leaf-chewer |
| Caterpillar (green, yellow legs) | Lepidoptera |  | sp.6 | Leaf-chewer |
| Caterpillar (green/ yellow longitudinal stripe) | Lepidoptera |  | sp.7 | Leaf-chewer |
| Caterpillar (green/ yellow oblique stripe) | Lepidoptera |  | sp.8 | Leaf-chewer |
| Caterpillar (large green) | Lepidoptera |  | sp.9 | Leaf-chewer |
| Caterpillar (orange tufts) | Lepidoptera | Lymantriidae | *Orgyia antiqua* | Leaf-chewer |
| Caterpillar (other totrix) | Lepidoptera | [Tortricidae](http://data.gbif.org/species/11316833) | sp.1 | Leaf-roller |
| Caterpillar (puss moth) | Lepidotera | Notodontidae | *Cerura vinula* | Leaf-chewer |
| Caterpillar (rose tortrix) | Lepidoptera | [Tortricidae](http://data.gbif.org/species/11316833) | *Archips rosana* | Leaf-roller |
| Caterpillar (zigzag) | Lepidotera | Notodontidae | *Notodonta ziczac* | Leaf-chewer |
| Click beetle (brown) | Coleoptera | Elateridae | sp.1 | Visitor |
| Cocoon (encased by yellow ‘net’) |  |  | sp.1 | Unknown |
| Cocoon-maker | Hymenoptera | Braconidae | *Apanteles* sp.1 | Visitor |
| Damsel bug (beige) | Hemiptera | Nabidae | sp.1 | Visitor |
| Egg (solitary, grey) |  |  | sp.1 | Egg |
| Egg (solitary, white) |  |  | sp.2 | Egg |
| Eggs (black, adaxial) |  |  | sp.3 | Egg |
| Eggs (cluster, green, tiny) |  |  | sp.4 | Egg |
| Eggs (cluster, beige/pink) |  |  | sp.5 | Egg |
| Eggs (cluster, grey) |  |  | sp.6 | Egg |
| Eggs (cluster, orange) |  |  | sp.7 | Egg |
| Eggs (cluster, white) |  |  | sp.8 | Egg |
| Eggs (silver) |  |  | sp.9 | Egg |
| Egg (solitary, blue) |  |  | sp.10 | Egg |
| Extra-floral nectary galler | [Acari](http://www.insectimages.org/browse/tax.cfm?order=291) | [Eriophyidae](http://www.insectimages.org/browse/tax.cfm?fam=641) | *Eriophyes diversipunctatus* | Gall-maker |
| Fly mine (white) | Diptera |  | sp.1 | Leaf-miner |
| Green leaf galler (adaxial) |  |  | sp.1 | Gall-maker |
| Lacewing | Neuroptera | Chrysopidae | *Chrysoperla carnea* | Visitor |
| Ladybird (7 spot) | Coleoptera | [Coccinellidae](http://en.wikipedia.org/wiki/Coccinellidae) | [*Coccinella*](http://en.wikipedia.org/wiki/Coccinella) *septempunctata* | Visitor |
| Ladybird (cream/black) | Coleoptera | [Coccinellidae](http://en.wikipedia.org/wiki/Coccinellidae) | *Propylea* sp.1 | Visitor |
| Ladybird (yellow/black) | Coleoptera | [Coccinellidae](http://en.wikipedia.org/wiki/Coccinellidae) | *Synharmonia* sp.1 | Visitor |
| Large round galler | Diptera | Cecidomyiidae | *Harmandia tremulae* | Gall-maker |
| Leaf hopper (green(black striped) | Hemiptera | Cicadellidae | sp.1 | Sap-sucker |
| Leaf hopper (green) | Hemiptera | Cicadellidae | sp.1 | Sap-sucker |
| Leaf beetle | Coleoptera | Chrysomelidae | *Phratora vitellinae* | Leaf-chewer |
| Leaf beetle | Coleoptera | Chrysomelidae | *Chrysomela populi* | Leaf-chewer |
| Leaf beetle | Coleoptera | Chrysomelidae | *Chrysomela tremulae* | Leaf-chewer |
| Leaf beetle | Coleoptera | Chrysomelidae | *Zeugophora* sp. 1 | Leaf-chewer / miner |
| Leaf beetle | Coleoptera | Chrysomelidae | *Phytodecta* sp.1 | Leaf-chewer |
| Leaf beetle (spotted) | Coleoptera | Chrysomelidae | *Cryptocephalus sexpunctatus* | Leaf-chewer |
| Leaf hopper (brown) | Hemiptera | Cicadellidae | sp.2 | Sap-sucker |
| Leaf hopper (pink) | Hemiptera | Cicadellidae | sp.3 | Sap-sucker |
| Leaf hopper (white) | Hemiptera | Cicadellidae | sp.4 | Sap-sucker |
| Leaf-cluster tier (loose roll) | Lepidoptera |  | sp.1 | Leaf-tier |
| Leaf-curl galler | Diptera | Cecidomyiidae | *Dasineura populeti* | Gall-maker |
| Leaf-mining fly | Diptera | Agromyzidae | Aulagromyza tremulae | Leaf-miner |
| Leaf-mining sawfly | Hymenoptera | Tenthredinidae | Heterarthrus ochropoda | Leaf-miner |
| Leaf-roller (half leaves) | Lepidoptera | Tortridcidae | sp.2 | Leaf-roller |
| Leaf-roller (rolls perpendicular to midrib) | Lepidoptera | Tortricidae | *Archips* sp.1 | Leaf-roller |
| Leaf-roller (two leaves) | Lepidoptera | Tortricidae | *Archips* sp.2 | Lea-roller |
| Leaf-rolling weevil | Coleoptera | Attelabidae | *Byctiscus betulae* | Leaf-roller |
| Leaf-rolling weevil | Coleoptera | Attelabidae | *Byctiscus populi* | Leaf-roller |
| Lepidopteran leaf-tying larva | [Lepidoptera](http://en.wikipedia.org/wiki/Lepidoptera) | [Drepanidae](http://en.wikipedia.org/wiki/Drepanidae) | *Tethea or* | Leaf-tier |
| Microlepidopteran miner | Lepidoptera | Gracillariidae | *Phyllocnistis labyrinthella* | Leaf-miner |
| Microlepidopteran miner | Lepidoptera | Gracillariidae | *Phyllocnistis unipunctella* | Leaf-miner |
| Petiole galler | Diptera | [Cecidomyiidae](http://www.insectimages.org/browse/tax.cfm?fam=98) | *Contarinia petioli* | Gall-maker |
| Red soldier beetle | Coleoptera | Cantharidae | *Rhagonycha fulva* | Visitor |
| Sawfly larva (green) | Hymenoptera | [Tenthredinidae](http://data.gbif.org/species/browse/provider/1/taxon/13143250/) | *Nematus* sp.1 | Visitor |
| Sawfly slug | Hymenoptera | [Tenthredinidae](http://data.gbif.org/species/browse/provider/1/taxon/13143250/) | *Caliroa tremulae* | Leaf-chewer |
| Shield bug | Hemiptera | Pentatomidae | sp.1 | Visitor |
| Small round galler | Diptera | Cecidomyiidae | *Harmandia gobuli* | Gall-maker |
| Spider (brown) | Araneae | Araneidae | *Araniella* sp.1 | Visitor |
| Spider (brown/cream) | Araneae | Araneidae | sp.1 | Visitor |
| Spider (green) | Araneae | Araneidae | sp.2 | Visitor |
| Spider egg sack (grey) | Araneae |  | sp.3 | Visitor |
| Spot galler (red) | [Acari](http://www.insectimages.org/browse/tax.cfm?order=291) | [Eriophyidae](http://www.insectimages.org/browse/tax.cfm?fam=641) | *Aceria varia* | Gall-maker |
| Spot galler (yellow) | [Acari](http://www.insectimages.org/browse/tax.cfm?order=291) | [Eriophyidae](http://www.insectimages.org/browse/tax.cfm?fam=641) | *Phyllocoptes populi* | Gall-maker |
| Unknown beetle (black, round) | Coleoptera |  | sp.1 | Unknown |
| Unknown beetle (black/red) | Coleoptera |  | sp.2 | Unknown |
| Unknown beetle (small, black/cream) | Coleoptera |  | sp.3 | Unknown |
| Unknown blister mine |  |  | sp.1 | Leaf-miner |
| Unknown blotch mine |  |  | sp.1 | Leaf-miner |
| Unknown gall-maker (white, abaxial gall) |  |  | sp.2 | Gall-maker |
| Unknown lacewing (large) | Neuroptera | Chrysopidae | sp.1 | Visitor |
| Unknown leaf-roller (leaf corner) |  |  | sp.1 | Leaf-roller |
| Unknown sawfly (imago) | Hymenoptera |  | sp.1 | Visitor |
| Unknown sawfly larva (brown/black) |  |  | sp.2 | Leaf-chewer |
| Unknown white cocoon (abaxial) |  |  | sp.1 | Unknown |
| Weevil | Coleoptera | Curculionidae | *Phyllobius maculicornis* | Leaf-chewer |
